# Timely Diagnosis of Cobalamin C Disease via Rapid Genome Sequencing in a Neonate With Severe Prenatally Detected Biventricular Dysfunction

**DOI:** 10.1155/crcc/2778022

**Published:** 2026-08-02

**Authors:** Yutaka Furuta, Samantha N. Walkin, Scott K. Ward, Rory J. Tinker, Rizwan Hamid, John A. Phillips, Rachel E. Harris, Thomas A. Cassini

**Affiliations:** ^1^ Department of Pediatrics, Division of Medical Genetics and Genomic Medicine, Vanderbilt University Medical Center, Nashville, Tennessee, USA, vanderbilt.edu; ^2^ Department of Medical Genetics and Genomics, Icahn School of Medicine at Mount Sinai, New York, New York, USA, mountsinai.org; ^3^ Department of Pediatrics, Division of Pediatric Cardiology, Vanderbilt University Medical Center, Nashville, Tennessee, USA, vanderbilt.edu

**Keywords:** biventricular dysfunction, cardiomyopathy, cobalamin C disease, intensive care, rapid genome sequencing

## Abstract

Cobalamin C (Cbl‐C) disease, the most common inborn error of cobalamin metabolism caused by biallelic pathogenic *MMACHC* variants, leads to multisystem involvement from methylmalonic acid and homocysteine accumulation. When diagnosed early, it is treatable. Rapid genome sequencing (GS) is becoming increasingly accessible and enables timely diagnosis and prompt treatment. We report a neonate who had severely decreased prenatal and postnatal biventricular function requiring inotropic support. Given the critically ill condition and unclear etiology, rapid GS was obtained and revealed homozygous pathogenic *MMACHC* variants, confirming a diagnosis of Cbl‐C disease. This prompted the early initiation of treatment with high‐dose hydroxocobalamin, betaine, levocarnitine, and folic acid. His cardiac function gradually improved, and he was discharged at 28 days of life. Critical care providers should suspect treatable inherited metabolic disorders in patients with unexplained presentations and recognize rapid GS as a powerful tool for early identification and treatment in critically ill patients.

## 1. Background

Cobalamin C (Cbl‐C) disease, also known as methylmalonic aciduria and homocystinuria, Cbl‐C type (MAHCC: OMIM #277400), is the most common inborn error of cobalamin metabolism [1, 2]. It is a disorder with autosomal recessive inheritance caused by variants in the *MMACHC* gene [3]. Cbl‐C defect impairs conversion of dietary cobalamin into its two metabolically active forms, methylcobalamin (MeCbl) and adenosylcobalamin (AdoCbl). These active forms are essential cofactors for the enzymes methionine synthase (MS) and methylmalonyl‐CoA mutase (MUT), respectively. Impaired MS leads to the accumulation of homocysteine (Hcy), and impaired MUT activity leads to the accumulation of methylmalonic acid (MMA).

This is typically detected initially on newborn screening (NBS) where C3 and methionine levels are measured on dried blood spots using tandem mass spectrometry. If elevated C3 and/or low methionine levels are detected, total Hcy and MMA levels will be measured as second tier tests [1]. A confirmatory diagnosis of Cbl‐C disease is typically established by biallelic pathogenic variants in *MMACHC* through molecular genetic testing. NBS is widely used in the United States for early detection of Cbl‐C disease [4].

Treatment of Cbl‐C disease includes supplementation of hydroxocobalamin, betaine, folic acid, and carnitine. In Cbl‐C disease, hydroxocobalamin is necessary as cyanocobalamin is ineffective [1]. Importantly, intramuscular, subcutaneous, or intravenous administration of hydroxocobalamin has been shown to be more effective than oral [1]. Betaine serves as the substrate for betaine homocysteine methyltransferase (BHMT) to provide an alternative pathway for Hcy remethylation that bypasses the MeCbl‐dependent pathway. Betaine is given at a dose of 250 mg/kg/day orally to children to lower Hcy and to increase methionine levels [1, 5]. Folic acid is also often used as adjunctive therapy to enhance the remethylation pathway. Carnitine supplementation may help prevent secondary carnitine deficiency by facilitating the excretion of propionyl groups, although its beneficial effects remain unproven. Unlike methylmalonic acidemia, no proven efficacy has been demonstrated with dietary protein restriction [1].

Cbl‐C disease is classified as early‐ or late‐onset [6]. The early‐onset form typically presents in the first year of life with neurological, ophthalmologic, hematological, renal, cardiac, vascular, pulmonary, and gastrointestinal manifestations [1, 6]. The clinical presentations of Cbl‐C disease are usually less acute compared with the classic forms of methylmalonic acidemia. The late‐onset form can present at varying ages, from childhood to adulthood. Cardiac involvement is a recognized complication of Cbl‐C disease [1, 7]. Structural heart defects have been reported in up to 50% of affected individuals [8]. Cardiomyopathy and left ventricular (LV) noncompaction have also been reported [7, 9–13]. One case of prenatally detected dilated cardiomyopathy has been reported [13]. Given that disease‐specific metabolic therapies are available, early diagnosis is essential for improving outcomes.

Cbl‐C disease is biochemically characterized by increased plasma total Hcy, low to normal plasma methionine, homocystinuria, and methylmalonic acidemia and aciduria. Levels of MMA in patients with Cbl‐C disease are usually lower than those seen in classical forms of methylmalonic aciduria caused by MUT deficiency [14]. Increased propionylcarnitine (C3) and a C3/C2 ratio can be seen in Cbl‐C disease, as well as methylmalonic acidemia and propionic acidemia. Therefore, total Hcy, methionine, and MMA, as well as acylcarnitine profiles and urine organic acids, are key diagnostic tools to identify Cbl‐C disease.

Here, we report the case of a neonate with early‐onset Cbl‐C disease who presented with severe unexplained prenatal and postnatal cardiac dysfunction. Confirmation of his diagnosis of Cbl‐C disease by rapid genome sequencing (GS) enabled prompt initiation of metabolic therapy, which included initiation of high‐dose hydroxocobalamin therapy.

## 2. Case Presentation

A 2450‐g male was born to a gravida 6 para 5, 42‐year‐old mother at 35 weeks and 2 days via cesarean delivery. He had marked impaired cardiac function and hydrops fetalis identified on fetal echocardiogram. Prenatal echocardiogram revealed severe biventricular dysfunction with right ventricular (RV) ejection fraction (RVEF) of 9% and left ventricular ejection fraction (LVEF) of 16%, RV hypertrophy, and right pleural effusion, with otherwise normal anatomic cardiac structures. Noninvasive prenatal testing and amniocentesis were declined. His mother was taking prenatal vitamins throughout this pregnancy. There was no history of maternal viral infections or gestational diabetes. There was no alcohol, tobacco, or illicit drug use during the pregnancy. Delivery was uncomplicated with an Apgar score of 7 at 1 and 9 at 5 min of life. After birth, he was immediately intubated and admitted to the neonatal intensive care unit (NICU) for management.

His family history revealed that his parents and five sibs were all in good health. There were no similar affected family members and no history of congenital heart diseases, cardiomyopathy, or inborn errors of metabolism. On arrival in the NICU, he was tachycardic (heart rate 178 beats/min), normal blood pressure (60/45 mmHg), respiratory rate (54 breaths/min), and oxygen saturation (94%) on mechanical ventilation with FiO_2_ 0.30. He was sedated. On physical examination, his length, weight, and head circumference were normal for age, and no dysmorphic features were noted. Cardiac auscultation revealed normal S1 and S2 with a soft 1/6 systolic murmur without a rub or gallop. Although his respiration was unlabored and his lungs were clear to auscultation, he had intermittent tachypnea. Abdominal examination was unremarkable with no hepatomegaly or splenomegaly. He was acrocyanotic with feet cool to touch, and capillary refill was 2–3 s.

An echocardiogram was obtained immediately after birth. It showed severely depressed biventricular function with LVEF of 26% and right ventricular fractional area change (RVFAC) of 18%, patent foramen ovale (PFO), large patent ductus arteriosus (PDA), and moderate RV dilation. His arterial blood gas showed lactic acidosis with pH of 7.21 and lactate of 4.0 mmol/L, brain natriuretic peptide (BNP) was 226 pg/mL (normal < 10–100 pg/mL), and ammonia level was 70 mcmol/L (normal < 80 *μ*mol/L).

He was immediately transferred to the cardiac intensive care unit for further management of his cardiac dysfunction. He was immediately started on an epinephrine infusion at 0.02 mcg/kg/min and milrinone at 0.25 mcg/kg/min. Epinephrine was discontinued on day of life 1 as he stabilized. Due to depressed LV function, he was started on enalapril and spironolactone. He was started on total parenteral nutrition with lipid supplementation. Infectious disease evaluation for potential viral myocarditis included testing for cytomegalovirus, parvovirus, Coxsackie virus, and adenovirus; all results were negative.

Genetics was consulted within 24 h of life and because of his critically ill condition and unknown etiology, an acylcarnitine profile, urine organic acids, free and total carnitine levels, and trio rapid GS were sent. His trio rapid GS was reported at 8 days of age. It showed a homozygous pathogenic variant in NM_015506.3 (MMACHC):c.271dup (p.Arg91fs). Since he had phenotypic overlap with Cbl‐C disease with his lactic acidosis, macrocytic anemia, thrombocytopenia, hydrops, and depressed biventricular function, it was felt that his molecular genetic testing results confirmed a diagnosis of Cbl‐C disease.

His initial NBS result became available on the same date as the rapid GS result (at 8 days of age) and reported elevated C3:C2 ratio of 0.97 (normal < 0.34), C3 of 7.26 mmol/L (normal < 6.85), MMA of 89.18 mmol/L (normal < 5.93), and 2‐methylcitric acid of 12.83 mmol/L (normal < 1.84) (Table 1). Acylcarnitine profile obtained at 4 days of age returned at 12 days of age, revealing elevated C3 of 12.62 mmol/L and elevated ratio of C3:C2 of 1.53 (Table 1). Urine organic acids sent at 3 days returned at 17 days of age showed elevated methylmalonic (1299 mmol/L), 3‐hydroxyproponic (754 mmol/L), methylcitric (78 mmol/L), and 2‐methylacetoacetic acids (8 mmol/L) (Table 1). Plasma total Hcy and methionine levels at 9 days of age were 241.9 mcmol/L (normal < 15) and 23 mcmol/L (normal 10–60), respectively (Table 1). These biochemical test results were congruent with the diagnosis of Cbl‐C disease.

**Table 1:** Biochemical testing (newborn screening, plasma amino acids, acylcarnitine profile, and urine organic acids) results.

Newborn screening	Plasma amino acids	Acylcarnitine profile	Urine organic acids
↑C3:C2 ratio	↑ total homocysteine	↑C3:C2 ratio	↑methylmalonic acid
↑total C3	Normal methionine	↑total C3	↑3‐hydroxyproponic acid
↑methylmalonic acid			↑methylcitric acid
↑2‐methylcitric acid			↑2‐methylacetoacetic acid

**Figure 1:**
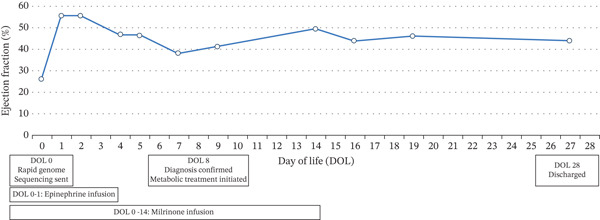
Left ventricular ejection fraction (LVEF) trend before and after starting metabolic treatment. Echocardiogram obtained on day of life (DOL) 0 showed severely depressed biventricular function with left ventricular ejection fraction (LVEF) of 26%. Inotropic support with epinephrine (DOL 0–1) and milrinone (DOL 0–14) was initiated. Trio rapid genome sequencing (GS) was sent on DOL 0 and resulted on DOL 8, confirming the molecular diagnosis of cobalamin C disease. Disease‐specific metabolic therapy was started immediately on DOL 8. Following the treatment, serial echocardiograms demonstrated a gradual improvement in his LVEF. The patient was discharged on DOL 28 with LVEF of 44%.

With the diagnosis of Cbl‐C disease established, treatment was instituted at 8 days of age with hydroxocobalamin 1 mg intramuscularly every 24 h, betaine 250 mg/kg/day divided every 8 hours, and levocarnitine 100 mg/kg/day divided every 12 h. His dosage of hydroxocobalamin was increased to 2 mg/kg daily because of a report that this dosage was protective of the eye disease and it was unclear whether cardiac function would respond to the traditional dosing (1 mg daily) [15]. Following this increase in hydroxocobalamin dosage at 8 days of age, serial echocardiograms demonstrated a gradual improvement in his LVEF, as shown in Figure 1. Following stabilization of his cardiac function, milrinone was discontinued at 14 days of age. Plasma total Hcy levels progressively decreased to 88.8, 56, 34.9, 16.6, and 17.3 mcmol/L at 10, 15, 19, 22, and 26 days of age, respectively. Plasma methionine levels remained within normal range at 15 days of age. At 26 days of age, plasma MMA was 0.59 mcmol/L (normal < 0.40). He was discharged at 28 days of age with LVEF improved to 44%.

After discharge, he continued follow‐up in genetics and cardiology clinics and remained on metabolic supplementations. Although he has remained clinically asymptomatic with stable biochemical parameters, his EF has gradually declined. At the most recent follow‐up, at 10 months of age, his cardiac function and biochemical profiles continue to be closely monitored.

## 3. Discussion

Here we report a neonate with severe biventricular cardiac dysfunction of unknown etiology that was diagnosed prenatally and confirmed postnatally. Rapid GS confirmed a diagnosis of early‐onset Cbl‐C disease, enabling the timely initiation of metabolic treatment.

Interestingly, cardiomyopathy is a recognized complication of Cbl‐C disease, but its cause is thought to be multifactorial. Proposed mechanisms of cardiomyopathy include direct toxicity from excess metabolites such as MMA and total Hcy, secondary carnitine deficiency, cardiotoxicity mediated through impaired oxidative phosphorylation, and dysfunction of the tricarboxylic acid (TCA) cycle, but the exact pathophysiology is poorly understood [1, 10, 16–18]. Consensus suggests that cardiac dysfunction presenting postnatally in patients with Cbl‐C disease resolves completely following hydroxocobalamin replacement therapy [13]. However, the timing and extent of the response of Cbl‐C disease‐associated cardiomyopathy to hydroxocobalamin supplementation have not been reported in detail.

In our patient′s case, high‐dose hydroxocobalamin (2 mg/kg/day) was initiated due to the reported increased risk for retinopathy associated with his identified variant [19], and the suggestion that this approach might ameliorate the development of this retinopathy [15]. The current international guideline recommends initiating in neonates with Cbl‐C disease with 1 mg per day of parenteral hydroxocobalamin, followed by individualized titration based on metabolic response [1]. However, there is little data on the optimal adjustment of hydroxocobalamin dosage and its long‐term data on its impact on cardiac phenotypes, despite several cases of high‐dose hydroxocobalamin treatment having been reported [15, 20–23]. In our case, cardiac function initially improved during hospitalization; however, the EF gradually declined after discharge despite ongoing metabolic therapies, including high‐dose hydroxocobalamin. Further studies are needed to evaluate the long‐term efficacy and safety of high‐dose hydroxocobalamin treatment in severe presentations.

Cardiomyopathy can be caused by a broad spectrum of genetic causes, including chromosomal anomalies (e.g., aneuploidies), single‐gene disorders, and inherited metabolic disorders (IMDs). Nonmetabolic single‐gene disorders associated with cardiomyopathy include pathogenic variants in *MYH7*, *MYBPC*3, *NEXN*, *ABCC9*, *ACTC1*, and *ACTN2*, as well as syndromic conditions such as RASopathies and neuromuscular disorders including Duchenne and Becker muscular dystrophies [24, 25]. IMDs associated with cardiomyopathy include organic acidemias, fatty acid oxidation disorders, mitochondrial disorders, lysosomal storage disorders, glycogen storage diseases, and congenital disorders of glycosylation [24, 25]. As demonstrated in our patient with Cbl‐C disease, early recognition of underlying IMD is critical because IMDs can be treatable and manageable when diagnosed in a timely manner and therapy is initiated promptly. His case emphasizes that metabolic etiologies should be considered in the differential diagnosis for unexplained perinatal cardiomyopathy. For this reason, massive parallel DNA sequencing is becoming an essential component of the diagnostic workup for IMDs in addition to traditional biochemical studies [26]. Rapid GS, a newer molecular diagnostic modality, further enhances diagnostic capability by dramatically shortening the time from clinical presentation to receipt of molecular diagnoses [27, 28]. Early implementation of rapid GS can facilitate prompt initiation of disease‐specific therapies, improve clinical outcomes, and reduce unnecessary procedures or delays in care. Although biochemical testing may provide important diagnostic clues within several days at centers with in‐house metabolic laboratories, such centers are limited. In many institutions, biochemical studies including acylcarnitine profile, urine organic acids, and MMA measurement, are performed at reference laboratories, resulting in longer turnaround times. As access to GS expands and turnaround times for results to return continue to improve, rapid GS is increasingly becoming an integral tool in the acute evaluation of critically ill patients with unexplained severe medical conditions.

## 4. Conclusions

IMDs including Cbl‐C disease can be treatable when specifically diagnosed in a timely manner. Critical care providers should maintain a high index of suspicion for these disorders in patients with unexplained clinical presentations to prevent delays in diagnosis and treatment. As rapid GS becomes increasingly accessible, critical care providers should recognize its role in facilitating early identification and treatment in critically ill patients with unexplained presentations. Further studies are needed to evaluate the efficacy and safety of high‐dose hydroxocobalamin treatment in severe presentations in Cbl‐C disease.

## Author Contributions

Conceptualization: Y.F. and T.A.C. Data curation: Y.F., S.K.W., and T.A.C. Formal analysis: Y.F. and T.A.C. Funding acquisition: N/A. Investigation: Y.F., S.K.W., and T.A.C. Methodology: Y.F., S.K.W., and T.A.C. Project administration: Y.F. and T.A.C. Resources: N/A. Software: N/A. Supervision: S.K.W., J.A.P., and T.A.C. Validation: Y.F., S.K.W., J.A.P., and T.A.C. Visualization: Y.F. Writing—original draft preparation: Y.F. Writing—review and editing: Y.F., S.N.W., S.K.W., R.J.T., R.H., J.A.P., R.E.H., and T.A.C.

## Funding

No funding was received for this manuscript.

## Consent

Informed consent for publication was obtained from the parent.

## Conflicts of Interest

The authors declare no conflicts of interest.

## Data Availability

Data sharing is not applicable to this article as no datasets were generated or analyzed during the current study.

## References

[1] Sloan J. L. , Carrillo N. , Adams D. , and Venditti C. P. , Adam M. P. , Bick S. , Mirzaa G. M. , Pagon R. A. , Wallace S. E. , and Amemiya A. , Disorders of Intracellular Cobalamin Metabolism, GeneReviews, 2008, University of Washington, Seattle, 1993–2025, 20301503. 20301503

[2] Huemer M. , Diodato D. , Schwahn B. , Schiff M. , Bandeira A. , Benoist J. F. , Burlina A. , Cerone R. , Couce M. L. , Garcia-Cazorla A. , la Marca G. , Pasquini E. , Vilarinho L. , Weisfeld-Adams J. D. , Kožich V. , Blom H. , Baumgartner M. R. , and Dionisi-Vici C. , Guidelines for Diagnosis and Management of the Cobalamin-Related Remethylation Disorders cblC, cblD, cblE, cblF, cblG, cblJ and MTHFR Deficiency, Journal of Inherited Metabolic Disease. (2017) 40, no. 1, 21–48, 10.1007/s10545-016-9991-4, 27905001. 27905001 PMC5203859

[3] Lerner-Ellis J. P. , Tirone J. C. , Pawelek P. D. , Doré C. , Atkinson J. L. , Watkins D. , Morel C. F. , Fujiwara T. M. , Moras E. , Hosack A. R. , Dunbar G. V. , Antonicka H. , Forgetta V. , Dobson C. M. , Leclerc D. , Gravel R. A. , Shoubridge E. A. , Coulton J. W. , Lepage P. , Rommens J. M. , Morgan K. , and Rosenblatt D. S. , Identification of the Gene Responsible for Methylmalonic Aciduria and Homocystinuria, cblC Type, Nature Genetics. (2006) 38, no. 1, 93–100, 10.1038/ng1683, 16311595. 16311595

[4] Ahrens-Nicklas R. C. , Whitaker A. M. , Kaplan P. , Cuddapah S. , Burfield J. , Blair J. , Brochi L. , Yudkoff M. , and Ficicioglu C. , Efficacy of Early Treatment in Patients With Cobalamin C Disease Identified by Newborn Screening: A 16-Year Experience, Genetics in Medicine. (2017) 19, no. 8, 926–935, 10.1038/gim.2016.214, 28151490. 28151490 PMC6082364

[5] Schiff M. , Benoist J. F. , Tilea B. , Royer N. , Giraudier S. , and Ogier de Baulny H. , Isolated Remethylation Disorders: Do Our Treatments Benefit Patients?, Journal of Inherited Metabolic Disease. (2011) 34, no. 1, 137–145, 10.1007/s10545-010-9120-8, 20490923. 20490923

[6] Martinelli D. , Deodato F. , and Dionisi-Vici C. , Cobalamin C defect: Natural History, Pathophysiology, and Treatment, Journal of Inherited Metabolic Disease. (2011) 34, no. 1, 127–135, 10.1007/s10545-010-9161-z, 20632110. 20632110

[7] Huemer M. , Scholl-Bürgi S. , Hadaya K. , Kern I. , Beer R. , Seppi K. , Fowler B. , Baumgartner M. R. , and Karall D. , Three New Cases of Late-Onset cblC Defect and Review of the Literature Illustrating When to Consider Inborn Errors of Metabolism Beyond Infancy, Orphanet Journal of Rare Diseases. (2014) 15, no. 9, 10.1186/s13023-014-0161-1. PMC4255922 25398587

[8] Profitlich L. E. , Kirmse B. , Wasserstein M. P. , Diaz G. A. , and Srivastava S. , High Prevalence of Structural Heart Disease in Children With cblC-Type Methylmalonic Aciduria and Homocystinuria, Molecular Genetics and Metabolism. (2009) 98, no. 4, 344–348, 10.1016/j.ymgme.2009.07.017, 19767224. 19767224

[9] Hjalmarsson C. , Backelin C. , Thoren A. , Bergh N. , Sloan J. L. , Manoli I. , Venditti C. P. , and Dellgren G. , Severe Heart Failure in a Unique Case of Cobalamin-C-Deficiency Resolved With LVAD Implantation and Subsequent Heart Transplantation, Molecular Genetics and Metabolism Reports. (2024) 39, no. 39, 101089, 10.1016/j.ymgmr.2024.101089, 38745823. 38745823 PMC11090888

[10] Liu Y. , Yang L. , Shuai R. , Huang S. , Zhang B. , Han L. , Sun K. , and Wu Y. , Different Pattern of Cardiovascular Impairment in Methylmalonic Acidaemia Subtypes, Frontiers in Pediatrics. (2022) 10, no. 10, 810495, 10.3389/fped.2022.810495, 35281223. 35281223 PMC8904414

[11] Tanpaiboon P. , Sloan J. L. , Callahan P. F. , McAreavey D. , Hart P. S. , Lichter-Konecki U. , Zand D. , and Venditti C. P. , Noncompaction of the Ventricular Myocardium and Hydrops Fetalis in Cobalamin C Disease, JIMD Reports. (2012) 10, 33–38, 10.1007/8904_2012_197, 23430797. 23430797 PMC3755569

[12] Xu D. , Zhang C. , Hao L. , Bi S. , Xue A. , Yuan L. , and Wang W. , Case Report: Dilated Cardiomyopathy as the Initial Presentation in an Adult With Late-Onset CblC Defect, Frontiers in Cardiovascular Medicine. (2026) 12, 1610295, 10.3389/fcvm.2025.1610295, 41658329. 41658329 PMC12872814

[13] De Bie I. , Nizard S. D. , and Mitchell G. A. , Fetal Dilated Cardiomyopathy: An Unsuspected Presentation of Methylmalonic Aciduria and Hyperhomocystinuria, cblC Type, Prenatal Diagnosis. (2009) 29, no. 3, 266–270, 10.1002/pd.2218, 19248038. 19248038

[14] Fowler B. , Leonard J. V. , and Baumgartner M. R. , Causes of and Diagnostic Approach to Methylmalonic Acidurias, Journal of Inherited Metabolic Disease. (2008) 31, no. 3, 350–360, 10.1007/s10545-008-0839-4. 18563633

[15] Scalais E. , Geron C. , Pierron C. , Cardillo S. , Schlesser V. , Mataigne F. , Borde P. , and Regal L. , Would, Early, Versus Late Hydroxocobalamin Dose Intensification Treatment, Prevent Cognitive Decline, Macular Degeneration and Ocular Disease, in 5 Patients With Early-Onset cblC Deficiency?, Molecular Genetics and Metabolism. (2023) 140, no. 3, 107681, 10.1016/j.ymgme.2023.107681, 37604084. 37604084

[16] Mc Guire P. J. , Parikh A. , and Diaz G. A. , Profiling of Oxidative Stress in Patients With Inborn Errors of Metabolism, Molecular Genetics and Metabolism. (2009) 98, no. 1-2, 173–180, 10.1016/j.ymgme.2009.06.007, 19604711. 19604711 PMC2915835

[17] de Keyzer Y. , Valayannopoulos V. , Benoist J. F. , Batteux F. , Lacaille F. , Hubert L. , Chrétien D. , Chadefeaux-Vekemans B. , Niaudet P. , Touati G. , Munnich A. , and de Lonlay P. , Multiple OXPHOS Deficiency in the Liver, Kidney, Heart, and Skeletal Muscle of Patients With Methylmalonic Aciduria and Propionic Aciduria, Pediatric Research. (2009) 66, no. 1, 91–95, 10.1203/PDR.0b013e3181a7c270, 19342984. 19342984

[18] Molema F. , Jacobs E. H. , Onkenhout W. , Schoonderwoerd G. C. , Langendonk J. G. , and Williams M. , Fibroblast Growth Factor 21 as a Biomarker for Long-Term Complications in Organic Acidemias, Journal of Inherited Metabolic Disease. (2018) 41, no. 6, 1179–1187, 10.1007/s10545-018-0244-6, 30159853. 30159853 PMC6327009

[19] Brooks B. P. , Thompson A. H. , Sloan J. L. , Manoli I. , Carrillo-Carrasco N. , Zein W. M. , and Venditti C. P. , Ophthalmic Manifestations and Long-Term Visual Outcomes in Patients With Cobalamin C Deficiency, Ophthalmology. (2016) 123, no. 3, 571–582, 10.1016/j.ophtha.2015.10.041, 26825575. 26825575 PMC5065014

[20] Kacpura A. , Frigeni M. , Gunther K. , and Farach L. , Clinical and Biochemical Outcomes in Cobalamin C Deficiency With Use of High-Dose Hydroxocobalamin in the Early Neonatal Period, American Journal of Medical Genetics Part A. (2022) 188, no. 6, 1831–1835, 10.1002/ajmg.a.62687. 35156754

[21] Higashimoto T. , Kim A. Y. , Ogawa J. T. , Sloan J. L. , Almuqbil M. A. , Carlson J. M. , Manoli I. , Venditti C. P. , Gunay-Aygun M. , and Wang T. , High-Dose Hydroxocobalamin Achieves Biochemical Correction and Improvement of Neuropsychiatric Deficits in Adults With Late Onset Cobalamin C Deficiency, JIMD Reports. (2020) 51, no. 1, 17–24, 10.1002/jmd2.12087, 32071835. 32071835 PMC7012733

[22] Matos I. V. , Castejón E. , Meavilla S. , O′Callaghan M. , Garcia-Villoria J. , López-Sala A. , Ribes A. , Artuch R. , and Garcia-Cazorla A. , Clinical and Biochemical Outcome after Hydroxocobalamin Dose Escalation in a Series of Patients With Cobalamin C Deficiency, Molecular Genetics and Metabolism. (2013) 109, no. 4, 360–365, 10.1016/j.ymgme.2013.05.007, 23746552. 23746552

[23] Olivieri G. , Greco B. , Cairoli S. , Catesini G. , Lepri F. R. , Orazi L. , Mallardi M. , Martinelli D. , Ricci D. , Simeoli R. , and Dionisi-Vici C. , Improved Biochemical and Neurodevelopmental Profiles With High-Dose Hydroxocobalamin Therapy in Cobalamin C Defect, Journal of Inherited Metabolic Disease. (2025) 48, no. 1, e12787, 10.1002/jimd.12787, 39152755. 39152755 PMC11670441

[24] Kohaut E. , Ader F. , Rooryck C. , Pelluard F. , Bonnière M. , André G. , Sauvestre F. , Roth P. , Khraiche D. , Bessières B. , Attié-Bitach T. , and Richard P. , Morphological and Genetic Causes of Fetal Cardiomyopathies, Clinical Genetics. (2023) 104, no. 1, 63–72, 10.1111/cge.14333, 37209000. 37209000

[25] Trakmulkichkarn T. , Ghadiry-Tavi R. , Fruitman D. , Niederhoffer K. Y. , Caluseriu O. , Lauzon J. L. , Wewala G. , Hornberger L. K. , Urschel S. , Conway J. , and McBrien A. , Clinical Presentation, Genetic Etiology and Outcome Associated With Fetal Cardiomyopathy: Comparison of Two Eras, Ultrasound in Obstetrics & Gynecology. (2022) 59, no. 3, 325–334, 10.1002/uog.23713, 34159662. 34159662

[26] Furuta Y. , Tinker R. J. , Hamid R. , Cogan J. D. , Ezell K. M. , Oglesbee D. , DeBerardinis R. J. , Phillips J. A. , and the Undiagnosed Diseases Network , A Review of Multiple Diagnostic Approaches in the Undiagnosed Diseases Network to Identify Inherited Metabolic Diseases, Orphanet Journal of Rare Diseases. (2024) 19, no. 1, 10.1186/s13023-024-03423-3, 39543639. PMC11566889 39543639

[27] Petrikin J. E. , Willig L. K. , Smith L. D. , and Kingsmore S. F. , Rapid Whole Genome Sequencing and Precision Neonatology, Seminars in Perinatology. (2015) 39, no. 8, 623–631, 10.1053/j.semperi.2015.09.009, 26521050. 26521050 PMC4657860

[28] Farnaes L. , Hildreth A. , Sweeney N. M. , Clark M. M. , Chowdhury S. , Nahas S. , Cakici J. A. , Benson W. , Kaplan R. H. , Kronick R. , Bainbridge M. N. , Friedman J. , Gold J. J. , Ding Y. , Veeraraghavan N. , Dimmock D. , and Kingsmore S. F. , Rapid Whole-Genome Sequencing Decreases Infant Morbidity and Cost of Hospitalization, NPJ Genomic Medicine. (2018) 4, no. 3, 10.1038/s41525-018-0049-4. PMC5884823 29644095

